# Archives of Scientific and Practical Medicine

**Published:** 1873-03

**Authors:** 


*Archives of Scientific and Practical Medicine.*

The January and February numbers of this new journal have been received. It is a first-class journal in every respect, and will, we doubt not, be prized by the profession. It is edited by Brown-Sequard, M. D., assisted by E. C. Seguin, M. D., and published by J. B. Lippincott & Co., Philadelphia. Price, $4 per annum.

